# On a three–dimensional model for the description of the passive characteristics of skeletal muscle tissue

**DOI:** 10.1007/s10237-022-01664-4

**Published:** 2022-12-22

**Authors:** Fabian Walter, Robert Seydewitz, Philipp Mitterbach, Tobias Siebert, Markus Böl

**Affiliations:** 1 Institute of Mechanics and Adaptronics, Technische Universität Braunschweig, D–38106 Braunschweig, Germany; 2 Mechanical Engineering, Eindhoven University of Technology, NLD–5612 Eindhoven, The Netherlands; 3 Institute of Sport and Motion Science, University of Stuttgart, D–70569 Stuttgart, Germany

**Keywords:** Skeletal muscle tissue model, Axial compression experiment, Semi–confined compression experiment, Parameter identification, Passive skeletal muscle behaviour

## Abstract

In this work, a three–dimensional model was developed to describe the passive mechanical behaviour of anisotropic skeletal muscle tissue. To validate the model, orientation–dependent axial ($0^\circ$, $45^\circ$, $90^\circ$) and semi–confined compression experiments (mode I, II, III) were performed on soleus muscle tissue from rabbits. In the latter experiments, specimen deformation is prescribed in the loading direction and prevented in an additional spatial direction, fibre compression at $0^\circ$ (mode I), fibre elongation at $90^\circ$ (mode II) and a neutral state of the fibres at $90^\circ$ where their length is kept constant (mode III). Overall, the model can adequately describe the mechanical behaviour with a relatively small number of model parameters. The stiffest tissue response during orientation–dependent axial compression ($-\,7.7\,\pm \,1.3$ kPa) occurs when the fibres are oriented perpendicular to the loading direction ($90^\circ$) and are thus stretched during loading. Semi–confined compression experiments yielded the stiffest tissue ($-\,36.7\,\pm \,11.2$ kPa) in mode II when the muscle fibres are stretched. The extensive data set collected in this study allows to study the different error measures depending on the deformation state or the combination of deformation states.

## Introduction

The mechanical behaviour of passive skeletal muscle tissue exhibits some unique characteristics that distinguish it from ordinary composite materials. Although the mechanical behaviour is well documented in the literature through numerous experimental investigations, the load–bearing mechanism of the complicated three–dimensional and hierarchical structure remains largely unknown. The interaction of muscle fibres and the collagenous, extracellular matrix (ECM) is responsible for the generation and transmission of active and passive forces and to resist various external loads. While the main function of muscle fibres is to generate active forces, the ECM serves as a connector to transfer load across different length scales (Böl et al. 2014, 2022). Experiments were performed on proteins (Linke et al. 1998; Baker et al. 2002; Pertici et al. 2018; Li et al. 2020), sarcomeres/myofibrils (Shalabi et al. 2017; de Souza Leite et al. 2017; Haeger and Rassier 2020; Lee et al. 2020; Haeger et al. 2020; Swist et al. 2020; Ward et al. 2020; Scellini et al. 2021; Marston 2022), fibres (Bartoo et al. 1997; Toursel et al. 2002; Fridén and Lieber 2003; Noonan et al. 2020; Böl et al. 2019, 2020, 2022), fibre bundles (Mutungi and Ranatunga 1996; Lieber et al. 2003; Meyer and Lieber 2011; Brown et al. 2012; Mathewson et al. 2014; Wood et al. 2014; Tamura et al. 2016; Wu et al. 2016; Noonan et al. 2020; Ward et al. 2020), and tissue level (Calvo et al. 2010; Morrow et al. 2010; Böl et al. 2012; Gras et al. 2012; Takaza et al. 2013b, a; Böl et al. 2014, 2016; Hashemi et al. 2020; Wheatley 2020; Leichsenring et al. 2021; Kohn et al. 2021; Kuravi et al. 2021; Böl et al. 2022) to characterise the mechanical properties at different scales. As previous studies at fibre (Böl et al. 2019, 2020) and tissue scale (Böl et al. 2014, 2016) have shown, individual fibres as well as fibres in combination with the ECM are capable of carrying compressive loads, suggesting a more comprehensive theory for the mechanical behaviour of muscle fibres and their contribution to the overall load response. Constitutive models originally developed for tissue materials such as the arterial wall (Holzapfel and Ogden 2010; Gasser 2018) or the heart muscle (Humphrey et al. 1990; Ambrosi and Pezzuto 2012), skeletal muscle (Martins et al. 1998; Johannson et al. 2000; Blemker et al. 2005; van Loocke et al. 2006; Röhrle and Pullan 2007; Odegard et al. 2008; Tang et al. 2009; Calvo et al. 2010; Ehret et al. 2011; Moerman et al. 2016), but also general formulations for fibre–reinforced soft tissue materials (Balzani et al. 2006; Ehret and Itskov 2007; Federico and Gasser 2010) use phenomenological approaches based on a hyperelastic strain energy function to predict the characteristic anisotropic load behaviour of soft tissue materials. Due to their relatively simple structure, they are easy to implement and adapt to different tissue types. A comprehensive overview of constitutive equations for various types of soft tissue materials are presented in the review article of Chagnon et al. (2015). To account for the anisotropic behaviour, additional so–called pseudo–invariants are often used in the constitutive relations, which take into account the deformation of the oriented fibres. Neglecting the ability of the fibre material to carry compressive loads, but also to account for different loading responses in tension and compression, the anisotropic contribution of the strain energy function during fibre compression is suppressed by using an additional on/off or heaviside function (Holzapfel et al. 2000; Federico and Gasser 2010). Model calibration often uses unloaded uniaxial compression tests in which the fibres are either compressed or stretched as a result of lateral expansion. There are only a few studies with so–called semi–confined compression experiments (Böl et al. 2014, 2016; Leichsenring et al. 2021). Here, the experimental setup allows, among other situations, the fibre length to be kept constant in order to investigate the anisotropic material behaviour under pressure more closely. In this sense, Böl et al. (2014) defined the fibre states under compression, tension, and at constant length as mode I, II, and III, respectively. Despite the differences, the experimental results of Böl et al. (2014) and Leichsenring et al. (2021) show different material responses for mode I and III, which illustrates the contribution of the muscle fibres to the load transfer of the entire tissue.

It is easy to see that all models with the anisotropic contribution disabled during compression are not able to distinguish between mode I and III , as the load transfer behaviour of the muscle fibres is not taken into account in these formulations. Therefore, it is not possible to find a set of material parameters that can predict the material response for all three fibre states. But also continuous approaches (Gasser et al. 2006) have difficulties in predicting the material behaviour. Consequently, the aim of the present work is to develop a simple phenomenological modelling approach at tissue level to predict the material response under different loading conditions and in different fibre directions within the limits of compression. Avoiding an on/off relationship allows a continuous transition between stress and compression for different fibre orientations. To verify the performance of the model, we apply the model to experimental studies under axial compression and semi–confined compression.

## Materials and methods

### Ethical approval

The study was exempted from ethical committee review according to national regulations (German Animal Welfare Act), as healthy, female domestic rabbits (*Oryctolagus cuniculus*) were obtained from a rabbit farm immediately after animal sacrifice.

### Tissue sample dissection and processing

Following Leichsenring et al. (2021), experiments were performed on muscle cubes taken from the soleus muscles of 18 female, white New Zealand rabbits (weight: 2700 ± 598 g, age: 75.2 ± 16.1 days). After transporting the animals in a cool box (at a temperature of $4^\circ$ C) to the laboratory, the soleus muscles were dissected out. The rabbit soleus has a simple unipennate muscle architecture with a mean optimal muscle fibre length of about 22 mm (Siebert et al. 2015), which allows cutting of tissue samples with fibre orientations parallel to the cube edge and at $45^\circ$. To stabilise the samples during cutting, the tissue was embedded in alginate, allowing the cutting of cubic samples with a mean edge length of 4.36 ± 0.56 mm. After preparation, the tissue samples were wrapped in cloths soaked with Dulbecco’s phosphate–buffered saline (DPBS) and stored in a climate chamber at $4^\circ$ C and 50% humidity. To warm the samples to a physiological temperature of $38.5^\circ$ C for the mechanical tests (Cooper et al. 1965; Siebert et al. 2017), they were removed from the climate chamber before the mechanical tests and placed in a bath of DPBS solution for 30 seconds.

### Experimental investigation on skeletal muscle tissue

**Fig. 1:**
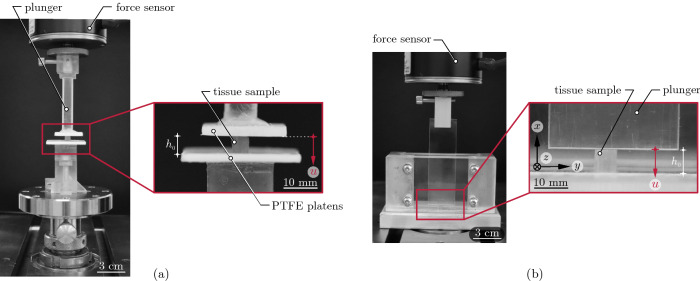
Deformation state, realised within this study: **a** Axial compression and **b** semi–confined compression experiments. All experiments were conducted with an axial testing machine (Zwick Z0.5, Zwick GmbH & Co. Ulm, Germany), equipped with a 5 N or 10 N load cell

As shown in previous work (e.g. Böl et al. 2013, 2014, 2016), the deformation state during muscle activation is highly inhomogeneous, indicating a complex load distribution within the muscle tissue where tension and compression are present. To gain a better understanding of the load–bearing mechanisms, in this study, we look at the material response of muscle tissue samples under axial and semi–confined compression for different muscle fibre orientations, see Fig. 1.

In the axial compression experiment (a), specimens are tested with their fibres oriented $0^\circ$, $45^\circ$, or $90^\circ$ with respect to the loading direction. In semi–confined compression (b), where specimen deformation is prescribed in the load direction and prevented in an additional spatial direction, fibre compression at $0^\circ$ (mode I), fibre elongation at $90^\circ$ (mode II) and a neutral state of the fibres at $90^\circ$(mode III) are examined. Table 1 shows an overview of the experiments performed in this study.

**Table 1:** Overview of the number of experiments realised in this study

	Axial compression			Semi–confined compression		
Orientation	$0^\circ$	$45^\circ$	$90^\circ$	I	II	III
No. of samples	12	16	14	14	15	15
Total	86					

#### Axial compression experiments

Following our previous investigation by Leichsenring et al. (2021), forty–two ($n=42$) cubic samples were subjected to orientation–dependent, axial compression (index: ac) testing up to 40% nominal compressive strain $\varepsilon$ at a strain rate of ${\dot{\varepsilon }}=0.5$ %$\hbox {s}^{-1}$. Specifically, three fibre orientations ($0^\circ$, $45^\circ$, $90^\circ$), to be defined as the angle between the fibre and the applied loading direction, were considered, see also Fig. 1a. While the displacement *u* of the upper platen was predefined, the resulting force *F* was measured and converted to mean engineering stress via $P=F/A_{ac}$ by division through the axial cross–sectional area $A_{ac}$, measured from a digital image which was recorded before testing. The compressive strain $\varepsilon =u/h_0$ was calculated from *u* and the undeformed sample height $h_0$. To diminish friction effects, the polytetrafluoroethylene (PTFE) platens were coated with silicone oil for lubrication (Böl et al. 2012). Additionally, a camera was positioned in front of the samples recording the sample’s transversal deformation.

#### Semi–confined compression experiments

Fourty–four ($n=44$) semi–confined (index: sc), orientation–dependent compression tests were performed using a custom–made testing device (Leichsenring et al. 2021). Three orientations, in which the fibres are aligned in the *x*–, *y*–, and *z*–directions, were considered, see Fig. 1b. These specimen orientations cause the fibre to be compressed (mode I), stretched (mode II), and held constant (mode III) during deformation. For details on the experimental setup, we refer to previous investigations (Böl et al. 2014, 2015a, 2016; Leichsenring et al. 2021).

To sample the specimens, they were carefully placed in the device, taking into account the fibre orientation. To reduce friction effects between the tissue and the device, the contact surfaces were sprayed with low viscosity silicone oil prior to the test, which had proven to be very effective in previous studies (Böl et al. 2014, 2015a, 2016; Leichsenring et al. 2021). Similar to the axial compression experiments, the tissue was compressed at a constant strain rate of ${\dot{\varepsilon }}=0.5$ %$\hbox {s}^{-1}$ to a maximum compression level of 40%. Again, the displacement *u* of the upper plunger was predefined, while the resulting force *F* was measured and converted to mean engineering stress $P=F/A_{sc}$, where $A_{sc}$ defines the axial cross–sectional area of the undeformed specimen. The axial strain $\varepsilon =u/h_0$ was calculated from *u* and the undeformed sample height $h_0$.

## Continuum mechanical framework of passive muscle tissue modelling

### Kinematics

Following general principles of continuum mechanics, we introduce ${\mathcal {B}}_0$ and ${\mathcal {B}}$ as the reference and current configurations of a body, in which the position of the particle is given by the vectors ${\varvec{X}}{}$ and ${\varvec{x}}{} = {\varvec{\varphi }}{}({\varvec{X}}{})$, respectively. Herein, the non–linear mapping of a material particle from the reference to the current configuration is represented by ${\varvec{\varphi }}{}({\varvec{X}}{}, t)$ and the corresponding deformation gradient ${\varvec{F}}{} = \nabla _X {\varvec{\varphi }}{}({\varvec{X}}{}, t)$. The Jacobian $J =\det {\varvec{F}}{}>0$ represents the volume change of the material particle and $\nabla _X(\bullet )$ indicates the spatial derivative with respect to the reference coordinates.

Accounting for the anisotropic behaviour of the tissue material, we introduce the fibre direction vector ${\varvec{M}}{}$ and the corresponding structural tensor

1 $$\begin{aligned} {\varvec{Z}}{} = {\varvec{M}}{} \otimes {\varvec{M}}{} \end{aligned}$$

in the reference configuration. The corresponding muscle fibre stretch

2 $$\begin{aligned} \lambda ^2 = I_4 = {\varvec{C}}{} : {\varvec{Z}}{} \end{aligned}$$

depends on the right Cauchy-Green tensor ${\varvec{C}}{}= {\varvec{F}}{}^T {\varvec{F}}{}$ and is in relation to the fourth invariant $I_4$.

Due to the high amount of water in living tissues, it is justified to assume incompressible material behaviour (Baskin and Paolini 1967). In computational mechanics it is often convenient to allow for slight volumetric changes and accordingly, to consider nearly incompressible material. The basic step for this procedure is a multiplicative split of the total deformation gradient into volumetric and isochoric parts according to Flory (1961) as

3 $$\begin{aligned} {\varvec{F}}{} = J^{1/3}\bar{{\varvec{F}}{}}, \end{aligned}$$

where $\bar{{\varvec{F}}{}}$ is the isochoric part of ${\varvec{F}}{}$. Accordingly, we obtain the isochoric counterpart of the right Cauchy–Green tensor as

4 $$\begin{aligned} \bar{{\varvec{C}}{}} = J^{-2/3}{\varvec{C}}{}=\bar{{\varvec{F}}{}}^T \bar{{\varvec{F}}{}} \end{aligned}$$

as well as the corresponding scalar valued first invariant

5 $$\begin{aligned} {\bar{I}}_1=\mathrm{tr}{\bar{{\varvec{C}}{}}}, \end{aligned}$$

representing an isotropic measure for the distortion of the matrix–fibre material.

### Constitutive equation for fibrous material under compression

Based on the results of recent experimental investigations, see Sects. 4.1 and 4.2, we propose a phenomenological modelling approach to describe the stress/strain behaviour during axial and semi–confined compression for different fibre orientations. For this purpose, we assume homogeneous deformations with perfectly aligned fibres in the direction of the main axes. In contrast to axial compression tests, the movement in semi–confined experiments is restricted in two directions, resulting in fibre being compressed (mode I, $\lambda <1$), stretched (mode II, $\lambda >1$), and held at a constant length (mode III $\lambda =1$). It is assumed that in mode I the compression counteracts the structural integrity of the fibre material, resulting in a weaker material response than in mode III, where the fibre length remains constant. To allow a smooth transition between the different modes and to predict the material behaviour for realistic deformations containing a combination of the three modes, a continuous strain energy function is proposed in the framework of hyperelasticity as

6 $$\begin{aligned} \psi (I_4, {\bar{I}}_1) = c_1\underbrace{e^{c_2(I_4-1)}}_{g(I_4)}\underbrace{(e^{c_3({\bar{I}}_1-3)}-1)}_{f({\bar{I}}_1)}+ K_{\mathrm{vol}}(J^2-1-2\ln J). \end{aligned}$$

Herein, the material parameters $c_1$ and $c_3$, $c_2$, and $K_\mathrm{vol}$ control the general load response, the anisotropic behaviour, and volume preservation, respectively. The bulk modulus $K_\mathrm{vol}$ was set to 5000 times the shear modulus of the tissue, resulting in nearly incompressible behaviour. Further, Equation (6) depends on the fourth invariant ($I_4$) and the isochoric part (${{\bar{I}}}_1$) of the first invariant, see also Remark 1.

The corresponding stress in terms of the 1st Piola-Kirchhoff stress follows from the derivation of the strain energy function with respect to the right Cauchy-Green tensor as

7 $$\begin{aligned} \begin{aligned}&{\varvec{P}}{} =2{\varvec{F}}{} \dfrac{\partial \psi }{\partial {\varvec{C}}}\\&\quad =2c_1 \bigg (\exp [c_2(I_4-1)+c_3({\bar{I}}_1-3)]\\&\qquad \left[ c_2 {\varvec{F}}{} {\varvec{M}}{} + c_3 J^{-2/3} \left( {\varvec{F}}{} - \frac{1}{3} \mathrm{tr}{\varvec{C}}{} {\varvec{F}}{}^{-T} \right) \right] \\&\quad -c_2 \exp [c_2(I_4-1)] {\varvec{FM}}{} \bigg ) + 2K_{\mathrm{vol}}(J^2 - 1){\varvec{F}}{}^{-T}. \end{aligned} \end{aligned}$$

The variable load behaviour for mode I, II, and III is mainly controlled by $g(I_4)$, since any deformation ${\varvec{F}}{} \ne {\varvec{I}}{}$ with $\det {\varvec{F}}{}>0$ leads to $f({\bar{I}}_1)>0$, where the particular formulation of $f({\bar{I}}_1)$ satisfies the stress–free reference condition of $\psi$. A common approach to modelling the anisotropic material behaviour is an additive split of the strain energy function into an isotropic and an anisotropic contribution that includes the fibre strain $I_4$ (e.g. Balzani et al. 2006; Moerman et al. 2016). However, deactivating the anisotropic component for $I_4<1$ excludes the fibres during compression, limiting the ability of the model formulation to predict the material response for all three modes and thus general deformations. For the current approach, $g(I_4)<1$ in mode I reduces the overall load response in the strain energy function $\psi$. In contrast, in mode II, $g(I_4)>1$ leads to an increase in $\psi$, while in mode III, the length of the muscle fibre remains constant and $g(I_4)=1$, leading to $\psi =\psi ({\bar{I}}_1,J)$. Note, Equation (6) is not convex due to fibre compression, which can lead to undesirable effects in a finite element simulation depending on the relationship between the material parameters $c_2$ and $c_3$ and the deformation present. However, the main objective of the present work was to find a simple structure for a strain energy function capable of capturing the different states of fibre compression.

#### Remark 1

(Volumetric/isochoric split of ${\varvec{F}}{}$) In Equation (6) and consequently in (7) we use the combination of ${\bar{I}}_1$ and $I_4$ to implement one–dimensional stresses in the fibre structure with respect to the reference configuration, which is consistent with the simplified representation that fibres act as one–dimensional truss elements. As already mentioned by Sansour (2008), the use of ${{\bar{I}}}_4$ does not imply a one–dimensional stress condition. Therefore, the use of ${{\bar{I}}}_4$ is limited to purely incompressible materials where the incompressibility condition is enforced by Lagrange multipliers (Nolan et al. 2014). In the present work, an alternative approach has been used that allows for slight volume changes using a penalty function and a static parameter.

### Optimisation scheme for material parameter identification

**Fig. 2:**
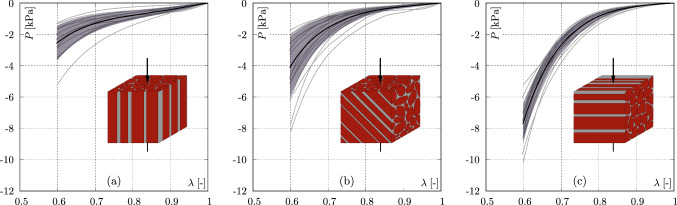
Stress–strain curves of the axial compression tests in dependence on the fibre orientation: **a** $0^\circ$, **b** $45^\circ$, and **c** $90^\circ$. Black curves indicate mean values, the shaded areas depict the standard deviation, and grey curves identify the single measurements

**Fig. 3:**
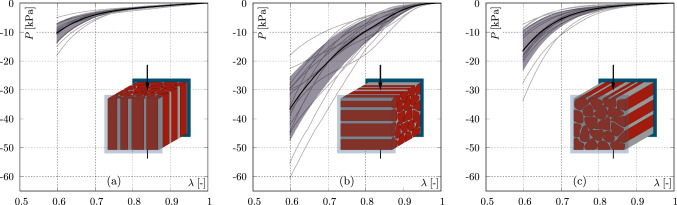
Stress–strain curves of the semi–confined compression tests in dependence on the different modes: **a** Mode I, **b** mode II , and **c** mode III. Black curves indicate mean values, the shaded areas depict the standard deviation, and grey curves identify the single measurements

To achieve successful identification of the material parameters, an inverse numerical optimisation procedure was introduced. In this process, a forward finite element analysis is coupled with an optimisation algorithm that determines the optimal set of parameters by adjusting the material parameters step by step to minimise the error between the measured and simulated responses, further denoted as inverse finite element method (iFEM). The basis for such parameter identification is, on the numerical side, the model approach for describing the mechanical behaviour of the anisotropic muscle tissue shown in Equation (6) and, on the experimental side, two deformation states (axial compression, semi–confined compression) described in Sect. 2.3 for three fibre orientations (axial compression: $0^\circ$, $45^\circ$, $90^\circ$ and semi–confined compression: mode I, mode II, mode III) each. Thus, the agreement between simulated and experimental data is evaluated using the objective function

8 $$\begin{aligned} {\mathcal {O}}({\varvec{p}}{})=\sum _{i=1}^{m} \sum _{j=1}^{n} \frac{1}{m \, n}\sqrt{\frac{(^i\!P^{\mathrm{sim}}_{j}-^i\!\!P^{\mathrm{exp}}_{j})^2}{^i\!P^{\mathrm{exp}}_{j}}}, \end{aligned}$$

which represents the combined relative square error of the included compression experiments. Herein, $^i\!P^{\mathrm{sim}}_j$ and $^i\!P^{\mathrm{exp}}_j$ are the orientation dependent simulated and measured stress values for every deformation increment, respectively. The use of silicone oil as a lubricant reduces frictional effects to a minimum and the resulting friction has been shown to be negligible (Böl et al. 2012). Therefore, friction effects are neglected in the FEM analyses within the scope of this study. Considering parallel ($0^\circ$) and perpendicular ($90^\circ$) oriented fibres, the axial and semi–confined compression tests within the friction limit lead to a homogeneous stress state, which allows the use of a single element for the FEM analysis. To achieve a compression of 40%, the FEM procedure performs 50 continuous load steps. For the subsequent adjustment of the strain measuring points of the calculation data, linear interpolation within measuring points is applied to both data sets. In addition, *m* takes into account the number of experiments (inform of stress–stretch relations) used simultaneously in the optimisation and *n* accounts for the numer of data sets used in the respective experiment. The following combinations were used in this study, see also Sect. 4.3: First, each of the 86 experiments was considered individually in the optimisation, resulting in $m=1$. In addition, the 3 directions of the axial compression and the semi–confined compression experiments (each in the form of mean value curves, see Figs. 2 and 3) were taken into account in the optimisation, resulting in a value of $m=3$ in each case. Finally, the mean stress–stretch relations of all directions and deformation states were considered simultaneously, so that $m=6$. To do this, mean value curves are created by first performing an interpolation for each data set and then calculating the mean stress at each sample point. To find an optimal parameter set in terms of the parameter vector ${\varvec{p}}{}=(c_1,c_2, c_3)^T$ the objective function was minimised, using the Nelder–Mead simplex algorithm (*fminsearch*, matlab$^{\circledR }$ R2018a) (Nelder and Mead 1965; Lagarias et al. 1998). Based on this algorithm, parameter sets were considered optimal when the objective function changed by less than the tolerance between two consecutive fitting steps.

## Results

### Axial compression experiments

Overall, the results of the axial compression tests show a non–linear, exponential material behaviour, independent of loading direction, see Fig. 2.

The maximum standard deviations consistently occur at the maximum compressive stress value. Expressed as a percentage of the corresponding mean value, i.e. s.d./mean $\times$100, the maximum standard deviations are less than 44%. Furthermore, it can be seen that the stiffest tissue response occurs for samples in which the fibres are oriented perpendicular to the loading direction, i.e., $90^\circ$. The softest response, on the other hand, is seen for tissue that is loaded parallel to the fibre orientation, i.e., $0^\circ$. There is a tendency for significant differences to exist between the $0^\circ$ and $90^\circ$ as well as between the $45^\circ$ and $90^\circ$ samples. It is particularly noticeable that the mean stress at 40% compression is –7.7±1.3 kPa for the sample set at $90^\circ$, and therefore about 3 times higher than for the sample set at $0^\circ$, to be –2.5±1.1 kPa, indicating strong anisotropic characteristics.

### Semi–confined compression experiments

Figure 3 illustrates the results of the orientation–dependent semi–confined compression experiments in form of mean stress–stretch curves and standard deviations.

Independent of the fibre orientation all curves are characterised by a clearly non–linear material response. For all orientations, the highest standard deviations are found at maximum compression. In general, these standard deviations are smaller than 43% of the corresponding mean value. Additionally, from Fig. 3 it is clear to see, that mode II, where the muscle fibres are stretched, shows with –36.7±11.2 kPa the stiffest response at 40% of compression. Here, mode III features the second stiffest response (–16.5±7.1 kPa), here the fibre length remains constant during deformation, followed by mode I (–10.4±3.5 kPa), where the fibres are compressed.

### Optimised material parameters

Using the parameter identification scheme described in Sect. 3.3, a wide variety of parameter sets were identified. In a first step, all experiments were optimised individually, resulting in 86 parameter sets, see Table 2.

**Table 2:** Material parameters and error measures determined from single, individual experiments

	Axial compression									Semi–confined compression									
Set no.	$0^\circ$			$45^\circ$			$90^\circ$			Mode I			Mode II			Mode III			${\mathcal {O}}$
	$c_1$ [kPa]	$c_2$ [–]	$c_3$ [–]	$c_1$ [kPa]	$c_2$ [–]	$c_3$ [–]	$c_1$ [kPa]	$c_2$ [–]	$c_3$ [–]	$c_1$ [kPa]	$c_2$ [–]	$c_3$ [–]	$c_1$ [kPa]	$c_2$ [–]	$c_3$ [–]	$c_1$ [kPa]	$c_2$ [–]	$c_3$ [–]	[–]
I	0.47	1.34	1.41	–	–	–	–	–	–	–	–	–	–	–	–	–	–	–	0.040
II	1.54	1.72	0.91	–	–	–	–	–	–	–	–	–	–	–	–	–	–	–	0.008
III	0.85	1.29	1.18	–	–	–	–	–	–	–	–	–	–	–	–	–	–	–	0.040
IV	0.99	0.75	0.82	–	–	–	–	–	–	–	–	–	–	–	–	–	–	–	0.027
V	0.62	1.16	0.84	–	–	–	–	–	–	–	–	–	–	–	–	–	–	–	0.004
VI	2.31	2.69	0.84	–	–	–	–	–	–	–	–	–	–	–	–	–	–	–	0.012
VII	3.40	0.01	0.13	–	–	–	–	–	–	–	–	–	–	–	–	–	–	–	0.058
VIII	6.13	1.26	0.20	–	–	–	–	–	–	–	–	–	–	–	–	–	–	–	0.038
IX	1.45	0.39	0.69	–	–	–	–	–	–	–	–	–	–	–	–	–	–	–	0.014
X	0.15	1.35	1.73	–	–	–	–	–	–	–	–	–	–	–	–	–	–	–	0.011
XI	0.83	1.47	0.71	–	–	–	–	–	–	–	–	–	–	–	–	–	–	–	0.051
XII	0.82	1.12	0.79	–	–	–	–	–	–	–	–	–	–	–	–	–	–	–	0.007
XIII	–	–	–	0.16	0.00	1.63	–	–	–	–	–	–	–	–	–	–	–	–	0.049
XIV	–	–	–	0.19	0.00	1.32	–	–	–	–	–	–	–	–	–	–	–	–	0.053
XV	–	–	–	0.14	0.00	2.33	–	–	–	–	–	–	–	–	–	–	–	–	0.087
XVI	–	–	–	0.12	1.09	1.66	–	–	–	–	–	–	–	–	–	–	–	–	0.017
XVII	–	–	–	0.88	2.70	0.95	–	–	–	–	–	–	–	–	–	–	–	–	0.052
XVIII	–	–	–	0.12	0.00	1.46	–	–	–	–	–	–	–	–	–	–	–	–	0.022
XIX	–	–	–	0.36	1.52	1.17	–	–	–	–	–	–	–	–	–	–	–	–	0.021
XX	–	–	–	0.20	0.00	1.84	–	–	–	–	–	–	–	–	–	–	–	–	0.034
XXI	–	–	–	0.14	0.00	2.51	–	–	–	–	–	–	–	–	–	–	–	–	0.063
XXII	–	–	–	0.64	0.23	1.26	–	–	–	–	–	–	–	–	–	–	–	–	0.040
XXIII	–	–	–	0.10	0.27	2.27	–	–	–	–	–	–	–	–	–	–	–	–	0.009
XXIV	–	–	–	0.16	0.00	1.95	–	–	–	–	–	–	–	–	–	–	–	–	0.040
XXV	–	–	–	0.11	0.00	2.33	–	–	–	–	–	–	–	–	–	–	–	–	0.052
XXVI	–	–	–	0.36	1.16	0.95	–	–	–	–	–	–	–	–	–	–	–	–	0.008
XXVII	–	–	–	0.11	0.00	1.84	–	–	–	–	–	–	–	–	–	–	–	–	0.016
XXVIII	–	–	–	0.32	0.00	1.43	–	–	–	–	–	–	–	–	–	–	–	–	0.219
XXIX	–	–	–	–	–	–	0.12	1.54	1.81	–	–	–	–	–	–	–	–	–	0.086
XXX	–	–	–	–	–	–	0.16	1.56	1.59	–	–	–	–	–	–	–	–	–	0.107
XXXI	–	–	–	–	–	–	0.33	1.57	0.96	–	–	–	–	–	–	–	–	–	0.130
XXXII	–	–	–	–	–	–	0.20	1.55	1.54	–	–	–	–	–	–	–	–	–	0.085
XXXIII	–	–	–	–	–	–	0.69	1.58	0.67	–	–	–	–	–	–	–	–	–	0.055
XXXIV	–	–	–	–	–	–	0.07	0.01	3.13	–	–	–	–	–	–	–	–	–	0.113
XXXV	–	–	–	–	–	–	0.40	1.58	1.07	–	–	–	–	–	–	–	–	–	0.152
XXXVI	–	–	–	–	–	–	0.19	1.56	1.49	–	–	–	–	–	–	–	–	–	0.125
XXXVII	–	–	–	–	–	–	0.11	1.62	1.97	–	–	–	–	–	–	–	–	–	0.092
XXXVIII	–	–	–	–	–	–	0.10	0.22	2.62	–	–	–	–	–	–	–	–	–	0.068
XXXIX	–	–	–	–	–	–	0.11	1.55	1.92	–	–	–	–	–	–	–	–	–	0.040
XL	–	–	–	–	–	–	0.37	1.54	0.90	–	–	–	–	–	–	–	–	–	0.041
XLI	–	–	–	–	–	–	0.22	1.56	1.56	–	–	–	–	–	–	–	–	–	0.103
XLII	–	–	–	–	–	–	0.52	1.57	0.98	–	–	–	–	–	–	–	–	–	0.121
XLIII	–	–	–	–	–	–	–	–	–	0.80	0.14	0.90	–	–	–	–	–	–	0.058
XLIV	–	–	–	–	–	–	–	–	–	1.56	0.92	0.84	–	–	–	–	–	–	0.078
XLV	–	–	–	–	–	–	–	–	–	0.90	0.27	0.72	–	–	–	–	–	–	0.075
XLVI	–	–	–	–	–	–	–	–	–	1.31	0.00	0.44	–	–	–	–	–	–	0.069
XLVII	–	–	–	–	–	–	–	–	–	4.60	1.09	0.40	–	–	–	–	–	–	0.037
XLVIII	–	–	–	–	–	–	–	–	–	9.33	0.14	0.07	–	–	–	–	–	–	0.092
XLIX	–	–	–	–	–	–	–	–	–	2.74	0.75	0.45	–	–	–	–	–	–	0.031
L	–	–	–	–	–	–	–	–	–	0.58	0.00	1.16	–	–	–	–	–	–	0.099
LI	–	–	–	–	–	–	–	–	–	1.50	0.00	0.37	–	–	–	–	–	–	0.070
LII	–	–	–	–	–	–	–	–	–	2.09	1.41	0.61	–	–	–	–	–	–	0.054
LIII	–	–	–	–	–	–	–	–	–	1.24	1.02	0.87	–	–	–	–	–	–	0.046
LIV	–	–	–	–	–	–	–	–	–	1.48	1.17	1.02	–	–	–	–	–	–	0.069
LV	–	–	–	–	–	–	–	–	–	1.70	0.86	0.62	–	–	–	–	–	–	0.045
LVI	–	–	–	–	–	–	–	–	–	1.82	0.00	0.40	–	–	–	–	–	–	0.100
LVII	–	–	–	–	–	–	–	–	–	–	–	–	33.68	0.01	0.06	–	–	–	0.119
LVIII	–	–	–	–	–	–	–	–	–	–	–	–	14.90	0.00	0.27	–	–	–	0.312
LIX	–	–	–	–	–	–	–	–	–	–	–	–	221.16	0.00	0.02	–	–	–	0.772
LX	–	–	–	–	–	–	–	–	–	–	–	–	214.93	0.18	0.02	–	–	–	1.058
LXI	–	–	–	–	–	–	–	–	–	–	–	–	2.07	0.06	0.89	–	–	–	0.567
LXII	–	–	–	–	–	–	–	–	–	–	–	–	202.29	0.00	0.02	–	–	–	0.478
LXIII	–	–	–	–	–	–	–	–	–	–	–	–	730.50	0.00	0.01	–	–	–	0.470
LXIV	–	–	–	–	–	–	–	–	–	–	–	–	223.62	0.42	0.01	–	–	–	1.228
LXV	–	–	–	–	–	–	–	–	–	–	–	–	4.12	0.38	0.31	–	–	–	0.829
LXVI	–	–	–	–	–	–	–	–	–	–	–	–	1596.57	0.00	0.00	–	–	–	0.814
LXVII	–	–	–	–	–	–	–	–	–	–	–	–	4.44	0.32	0.29	–	–	–	0.470
LXVIII	–	–	–	–	–	–	–	–	–	–	–	–	223.56	0.17	0.02	–	–	–	0.869
LXIX	–	–	–	–	–	–	–	–	–	–	–	–	6.71	0.19	0.38	–	–	–	0.403
LXX	–	–	–	–	–	–	–	–	–	–	–	–	11.20	0.01	0.26	–	–	–	0.221
LXXI	–	–	–	–	–	–	–	–	–	–	–	–	2.48	0.24	0.57	–	–	–	0.176
LXXII	–	–	–	–	–	–	–	–	–	–	–	–	–	–	–	4.07	2.95	0.16	0.115
LXXIII	–	–	–	–	–	–	–	–	–	–	–	–	–	–	–	0.61	0.99	1.53	0.253
LXXIV	–	–	–	–	–	–	–	–	–	–	–	–	–	–	–	0.42	0.67	1.28	0.090
LXXV	–	–	–	–	–	–	–	–	–	–	–	–	–	–	–	0.39	1.42	1.44	0.144
LXXVI	–	–	–	–	–	–	–	–	–	–	–	–	–	–	–	1.22	1.36	0.81	0.224
LXXVII	–	–	–	–	–	–	–	–	–	–	–	–	–	–	–	0.58	0.35	0.82	0.078
LXXVII	–	–	–	–	–	–	–	–	–	–	–	–	–	–	–	0.74	1.34	1.12	0.134
LXXIX	–	–	–	–	–	–	–	–	–	–	–	–	–	–	–	0.63	0.47	0.86	0.050
LXXX	–	–	–	–	–	–	–	–	–	–	–	–	–	–	–	0.13	2.12	1.58	0.067
LXXXI	–	–	–	–	–	–	–	–	–	–	–	–	–	–	–	0.75	0.30	1.00	0.102
LXXXII	–	–	–	–	–	–	–	–	–	–	–	–	–	–	–	1.13	0.73	1.07	0.251
LXXXIII	–	–	–	–	–	–	–	–	–	–	–	–	–	–	–	0.23	0.10	1.76	0.172
LXXXIV	–	–	–	–	–	–	–	–	–	–	–	–	–	–	–	0.17	1.58	1.41	0.085
LXXXV	–	–	–	–	–	–	–	–	–	–	–	–	–	–	–	0.24	1.11	1.49	0.247
LXXXVI	–	–	–	–	–	–	–	–	–	–	–	–	–	–	–	0.57	1.02	1.18	0.125
Mean	1.63	1.21	0.85	0.26	0.44	1.68	0.26	1.36	1.59	2.26	0.56	0.63	232.82	0.13	0.21	0.79	1.10	1.17	0.17
S.D	1.60	0.64	0.43	0.21	0.76	0.48	0.18	0.51	0.66	2.18	0.50	0.29	408.84	0.15	0.25	0.93	0.72	0.39	0.24

Within a deformation state, these parameter sets show similar parameter values overall. Only in mode II parameter values deviate decisively. This is also evident in the error measures. While these are very low overall, they increase significantly in mode II.

Since the identified individual parameter sets as described above only inadequately describe the mechanical behaviour of all experiments, parameters from further experimental set (combinations) were identified. Thus, material parameters were identified only from the (i) axial compression experiments ($0^\circ$, $45^\circ$, $90^\circ$, i.e. related to Equation (8) $m=3$), (ii) from the semi–confined compression experiments (mode I, mode II, mode III, i.e. $m=3$), related to Equation (8) $m=3$), and (iii) from all axial compression experiments ($0^\circ$, $45^\circ$, $90^\circ$) and semi–confined compression experiments (mode I, mode II, mode III). Thus $m=6$ in Equation (8). The latter case (iii) represents the most realistic situation, since on average the parameters for all experiments should be adequately represented here. The results of the three cases are shown in Table 3.

**Table 3:** Material parameters and error measures determined from various experiments/experiment combinations

		Parameter set ${\varvec{p}}{}$			
Experimental set	Considered deformation state(s)	$c_1$ [kPa]	$c_2$ [–]	$c_3$ [–]	${\mathcal {O}} ({\varvec{p}}{})$
(i)	Axial compression ($0^\circ$, $45^\circ$, $90^\circ$)	0.24	0.70	1.56	0.122
(ii)	Semi–confined compression (modes I, II, III)	1.39	0.52	0.66	0.512
(iii)	(i) & (ii)	0.39	0.53	1.27	0.473

Experimental set (i) yields the lowest error with ${\mathcal {O}}=0.122$. The highest error, with ${\mathcal {O}}=0.512$, is calculated for (ii) and especially the parameters $c_1$ and $c_3$ deviate strongly from those in parameter set (i). Experimental set (iii), with an error measure of ${\mathcal {O}}=0.473$, lies between cases (i) and (ii) and the parameters are similar in magnitude to those of case (i).

**Fig. 4:**
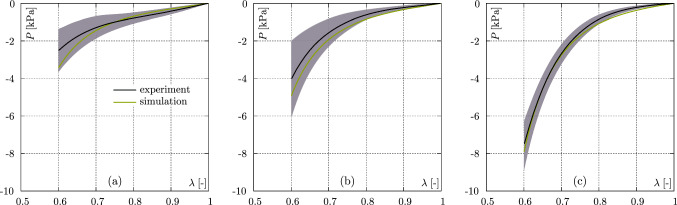
Comparison of the data from the axial compression experiments with the model response (with parameters from the experimental set (iii), see Table 3) for **a** $0^\circ$, **b** $45^\circ$, and **c** $90^\circ$. Green curves illustrate the model response, black curves indicate the mean value of the experimental data, and the shaded areas depict the standard deviation

Figure 4 shows the model response for experimental set (iii), providing the most meaningful parameters, compared to the axial compression experiments. Basically, the model can reproduce all three directions of the deformation state well. While the model for the $0^\circ$ direction shows a slightly too stiff behaviour beyond a stretching value of approximately 0.75, the model for the $45^\circ$ specimens generally shows a slightly stiffer material response. The $90^\circ$ model response, on the other hand, shows very good agreement with the experimental response over the entire stretching range.

**Fig. 5:**
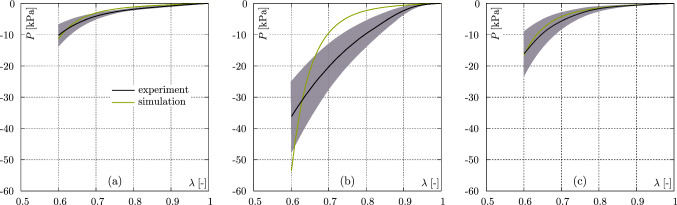
Comparison of the data from the semi–confined compression experiments with the model response (with parameters from the experimental set (iii), see Table 3) for **a** mode I, **b** mode II, and **c** mode III. Green curves illustrate the model response, black curves indicate the mean value of the experimental data, and the shaded areas depict the standard deviation

Figure 5 shows the model prediction for the semi–confined compression experiments using the same material parameters as for the axial compression simulations shown in Figure 4.

While the model can reproduce the tissue response for mode I and III very well, the model for mode II deviates significantly by overestimating the progressive increase in the stress response. The model shows a too soft response up to an elongation value of approximately 0.72. Compared to the experimental data, a stronger curvature of the stress–strain relationship can also be observed.

## Discussion

### Mechanical muscle tissue characteristics

To mechanically characterise the passive behaviour of the muscle tissue, in a first step orientation–dependent axial compression tests were performed, cf. Fig. 2. The stiffest tissue response (–7.7 ± 1.3 kPa) occurs when the fibres are oriented perpendicular to the loading direction ($90^\circ$) and are thus stretched during loading. Comparing the different fibre orientations, the mean stress for the $90^\circ$ orientation is 188% and 303% larger compared to the samples with a $45^\circ$ fibre orientation (–4.1 ± 2.0 kPa) and with a $0^\circ$ fibre orientation (–2.5 ± 1.1 kPa), respectively. This behaviour agrees well with some studies carried out on muscle tissue from pigs (van Loocke et al. 2006), rabbits (Böl et al. 2012), or chickens (Mohammadkhah et al. 2016), in which the passive properties of muscle tissue were determined as a function of fibre orientation. The anisotropic material behaviour of the muscle tissue was also reported by Pietsch et al. (2014) and Simms et al. (2016), investigating compressive loading of porcine muscle tissue in two different directions ($0^\circ$ and $90^\circ$). Both studies also determined lower stresses when the fibres are oriented in line to the loading direction.

In a second step, semi–confined compression experiments were performed to describe the mechanical properties of the muscle tissue. The stiffest tissue response occurs with –36.7 ± 11.2 kPa when the muscle fibres are stretched, i.e. mode II, see Fig. 3. This observation is consistent with tissue measurements performed on biceps femoris muscles of female domestic pigs (Böl et al. 2014, 2016) and soleus muscle of rabbits (Leichsenring et al. 2021). For mode I, the tissue response in the present study is softest at –10.4 ± 3.5 kPa, which is consistent with the study by Leichsenring et al. (2021), which also sampled soleus muscle tissue from rabbits. In contrast, Böl et al. (2014) and Böl et al. (2016) found the lowest stresses when the fibre lengths remained constant during the compression test, i.e., at mode III. The reason for the different mechanical results between the aforementioned studies is thought to be the ratio of fibres and ECM in the specific muscle tissue. For example, it is known that skeletal muscles within an animal or between different species differ both in their ECM (Gillies and Lieber 2011) and in the intracellular passive muscle structures (e.g. titin) (Prado et al. 2005). In addition, the ECM structure and amount changes with age (Ramaswamy et al. 2011; Leichsenring et al. 2021) and disease (Lieber and Ward 2013). These factors can lead to different directional behaviour of the muscle tissue under compression. The main aim of Böl et al. (2014) was to analyse the load transfer mechanisms between fibres and ECM. Since the muscles of different animals cited here may differ in their structure and thus in the relationship between fibres and ECM, the mechanical responses also vary. Furthermore, Böl et al. (2014) and Böl et al. (2016) describe significant differences between all three modes, which can also be confirmed in the present study and thus reveals information about the load transfer behaviour of the muscle fibres.

### Comparison with existing muscle models

As described in Sect. 4.3, the model proposed in this study is able to satisfactorily reproduce the deformation states shown here. However, to place this result, and thus the present muscle model, in the landscape of existing muscle models, the proposed material model formulation is compared with other skeletal muscle models. For the comparison given here, the typical models published in the field of passive muscle modelling were used, assuming ideal incompressibility for all models for simplicity. To evaluate the overall performance of the model, Table 4 lists the error values with respect to the deformation states, the model’s ability to produce higher stresses in mode III than in mode I, and the number of material parameters used in the corresponding model (last column).

**Table 4:** Error ${\mathcal {O}}({\varvec{p}}{})$ of the different material models when compared with the experimental data, model ability to produce higher stresses in mode III than in mode I (✓: distinction between mode III and mode I, ✗: no distinction between mode III and mode I), and the number of material parameters used in the corresponding model

	Error ${ {\mathcal O} ({\varvec{p}}{})}$								
Reference	$0^\circ$	$45^\circ$	$90^\circ$	Mode I	Mode II	Mode III	Mean	$P_{\mathrm{I}}<P_{\mathrm{III}}$	No. model parameters
Blemker et al. (2005)	0.89	0.24	0.61	1.21	0.45	0.67	0.68	✓	6
Calvo et al. (2010)	0.15	0.31	0.51	0.50	0.59	0.69	0.46	✗	5
Ehret et al. (2011)	0.33	0.37	0.16	0.36	1.74	0.39	0.56	✓	5
Johannson et al. (2000)	0.60	0.24	0.37	0.04	1.46	0.54	0.54	✗	4
Martins et al. (1998)	0.74	0.31	0.50	0.16	1.63	0.70	0.67	✗	4
Moerman et al. (2016)	0.23	0.28	0.46	0.60	0.59	0.77	0.49	✗	3
Odegard et al. (2008)	0.34	0.03	0.88	0.26	1.71	0.20	0.57	✓	4
Röhrle and Pullan (2007)	0.15	0.30	0.51	0.51	0.44	0.70	0.43	✗	6
Mean	0.43	0.26	0.50	0.46	1.08	0.58			
Present model	0.17	0.29	0.20	0.38	1.58	0.23	0.48	✓	3

As shown in the penultimate column, only the present approach, as well as the models of Blemker et al. (2005), Odegard et al. (2008), and Ehret et al. (2011) are basically able to generate higher stresses in mode III than I. Thereby, the stress responses of the model of Ehret et al. (2011) agree well with the experimental data.

**Fig. 6:**
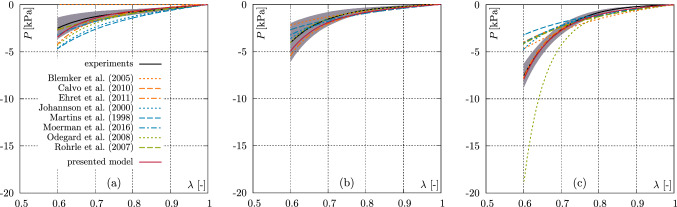
Comparison of the data from the axial compression experiments with existing models for **a** $0^\circ$, **b** $45^\circ$, and **c** $90^\circ$. Coloured curves illustrate the model responses, black curves indicate the mean value of the experimental data, and the shaded areas depict the standard deviation

**Fig. 7:**
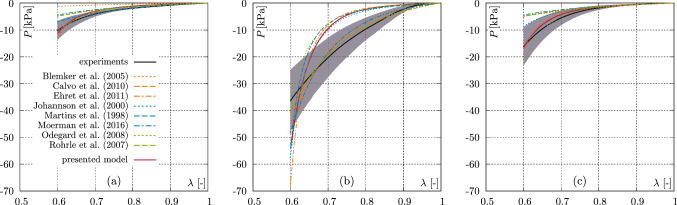
Comparison of the data from the semi–confined compression experiments with existing models for **a** mode I, **b** mode II, and **c** mode III. Coloured curves illustrate the model responses, black curves indicate the mean value of the experimental data, and the shaded areas depict the standard deviation

Figure 6 shows smaller deviations for axial compression and larger deviations for mode II in case of the semi–confined experiments, see Fig. 7b. In particular, the stresses at 40% compression are significantly higher than the maximum stresses produced by the present model in mode II. Overall, both models can reproduce the stress–stretch behaviour of the semi–confined deformation state. However, the present approach shows a slightly better overall performance. Although the model of Odegard et al. (2008) produces higher stresses in mode III than in mode I, the accuracy of the model prediction is limited. E.g., the stress response of the model fits very well for axial compression in the case of $45^\circ$ oriented fibres, but clearly deviates in case of the $0^\circ$ fibre orientation, which becomes even clearer for $90^\circ$ fibre orientation, see Fig. 6. Also for mode II the model shows clear deviations from the experimental data, see Fig. 7b. Within the models able to generate higher stresses in mode III than in mode I, the approach by Blemker et al. (2005) generates the highest error (mean value ${\mathcal {O}}({\varvec{p}}{}) = 0.68$). The model is able to reproduce mode II very well compared to the other models. For all other deformation states, rather larger deviations can be seen. In particular, it should be mentioned that the model does not provide any stress in the case of the axial compression test ($0^\circ$ fibre orientation), which is related to the model formulation.

For more detailed information, the interested reader is referred to Blemker et al. (2005).

While the stress responses, whose deviations from the experimental data in the form of error measures, are a characteristic, the number of parameters used by a model is also an important indicator of its quality. In the last column of the Table 4 the parameter numbers are given. While the model presented here uses 3 parameters, the approaches of Blemker et al. (2005), Odegard et al. (2008) and Ehret et al. (2011) use 6, 4, and 5 parameters respectively, which underlines the quality of the model presented here. The models of Röhrle and Pullan (2007), Moerman et al. (2016), and Calvo et al. (2010) produce the lowest mean error values. However, due to the Heaviside formulation to switch off the fibre contribution for $I_4<1$, the models do not distinguish between mode III and mode I. This further results in the model responses of these three models being identical for modes I and III, as they only provide the tissue matrix response. Overall, the presented model shows a good performance between the prediction of the stress and stretch behaviour of the fibres for different fibre orientations under compressive loading. Also in comparison to the modelling concepts by Blemker et al. (2005), Odegard et al. (2008), and Ehret et al. (2011), the model shows good performance resulting in the lowest mean error of ${\mathcal {O}}({\varvec{p}}{}) = 0.48$.

## Conclusions

The experimental basis for this study is an earlier experimental campaign (Leichsenring et al. 2021), providing comprehensive data on the muscle tissue of rabbit soleus muscles. Two orientation–dependent deformation states, namely axial and semi–confined compression, were realised. The orientation–dependent compression experiments show that the generated stresses increase from $0^\circ$ over $45^\circ$ up to $90^\circ$ fibre orientation. In the case of the semi–confined compression experiments, mode I shows the lowest and mode II the stiffest tissue response, with mode III in between. In this work we developed a phenomenologically motivated material formulation which can predict the stresses of the previously described deformation states with good agreement. A special feature here is the fact that mode III is stiffer than mode I in the semi–confined experiments. The proposed model contributes to a better understanding of the load transfer mechanisms between fibres and ECM (Böl et al. 2014, 2015b). Furthermore, it could lead to improved predictions of models of human muscles under compression, which are used e.g. in rehabilitation engineering, impact biomechanics, or for the simulation of surgical interventions (Guccione et al. 2001; van Rooij et al. 2003; Linder–Ganz et al. 2009; Siebert et al. 2018). While the model developed here can describe this behaviour, not all models in the literature are able to do so. Overall, the model presented here shows a good performance and describes the deformation states shown here with only three model parameters in a good way.

## Data Availability

Data and materials are available within this manuscript.
